# Incentives for new antibiotics: the Options Market for Antibiotics (OMA) model

**DOI:** 10.1186/1744-8603-9-58

**Published:** 2013-11-07

**Authors:** David M Brogan, Elias Mossialos

**Affiliations:** 1 Department of Orthopaedic Surgery, Mayo Clinic, Rochester, MN 55905, USA; 2 Department of Social Policy, London School of Economics and Political Science, London WC2A 2AE, United Kingdom

**Keywords:** Antimicrobial resistance, Antibiotic development, Options Market for Antibiotics, Incentive mechanisms for new antibiotics

## Abstract

**Background:**

Antimicrobial resistance is a growing threat resulting from the convergence of biological, economic and political pressures. Investment in research and development of new antimicrobials has suffered secondary to these pressures, leading to an emerging crisis in antibiotic resistance.

**Methods:**

Current policies to stimulate antibiotic development have proven inadequate to overcome market failures. Therefore innovative ideas utilizing market forces are necessary to stimulate new investment efforts. Employing the benefits of both the previously described Advanced Market Commitment and a refined Call Options for Vaccines model, we describe herein a novel incentive mechanism, the Options Market for Antibiotics.

**Results:**

This model applies the benefits of a financial call option to the investment in and purchase of new antibiotics. The goal of this new model is to provide an effective mechanism for early investment and risk sharing while maintaining a credible purchase commitment and incentives for companies to ultimately bring new antibiotics to market.

**Conclusions:**

We believe that the Options Market for Antibiotics (OMA) may help to overcome some of the traditional market failures associated with the development of new antibiotics. Additional work must be done to develop a more robust mathematical model to pave the way for practical implementation.

## Background

It is well recognized that there is an impending global crisis in novel antibiotic development. In a 2004 survey of publicly disclosed new molecular entities by the world’s 15 largest pharmaceutical manufacturers, only 1.6% were antibacterial agents [1]. The trend has only worsened in recent years, with the former head of the European Medicine’s Agency (EMA) Thomas Lonngren, decrying in 2010, the existence of “a gap of 5 years without research into new antibiotics” [2]. While dramatic, this underscores the perceived underfunding of antibiotic research at a time when antibiotic resistant pathogens have become increasingly prevalent across the globe. Attention has recently focused on the ESKAPE pathogens – a collection of microbial organisms known for their particular virulence and resistance to current antimicrobial regimens. The ESKAPE pathogens include: *Enterococcus faecium, Staphylococcus aureus, Klebsiella pneumoniae, Acinetobacter baumanni, Pseudomonas aeruginosa,* and *Enterobacter* species [3]. The burden of these diseases disproportionately affects hospitalized patients – it is estimated that annual hospital deaths in the European Union alone from drug resistant versions of the above pathogens top 25,000 patients, with costs totaling more than EUR €900 million [4]. In the US, the total annual cost of antibiotic resistance has been estimated at US $26 billion [5], and the direct mortality from antibiotic resistance infections has been reported at 23,000 deaths annually [6].

**Figure 1:**
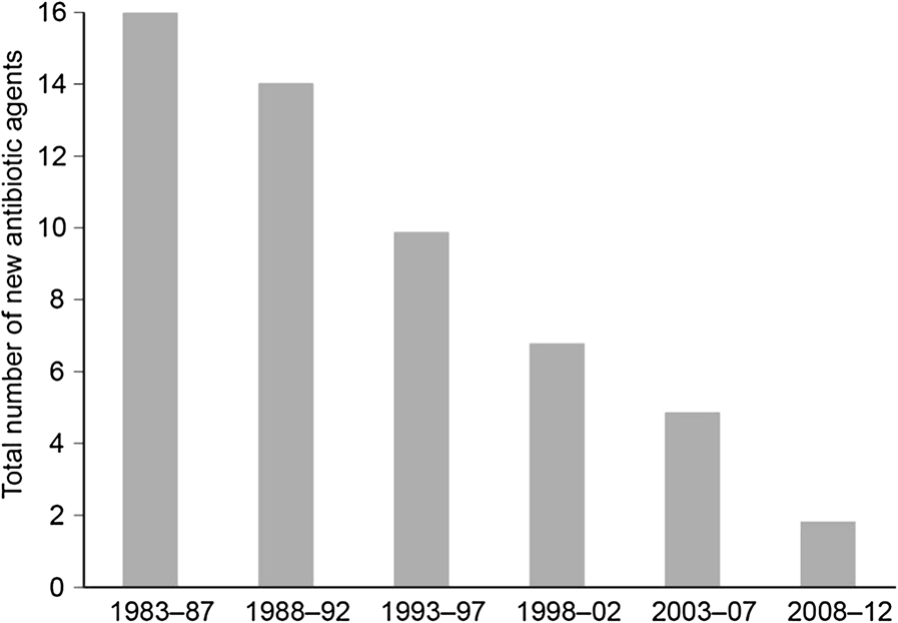
**Number of New Molecular Entities Approved by the FDA per 5 year period through March 2011. ** Source: [10]. By permission of Oxford University Press/on behalf of the Infectious Disease Society of America. Any reuse requires permission from Oxford University Press.

As a class of drugs, antibiotics have several unique properties which make them less profitable and therefore less attractive to corporate investment. To begin with, many antibiotics are prescribed for a relatively short course, anywhere from 3 days to 2 weeks, as compared to a course of years or decades for anti-hypertensive or cholesterol medications. Contributing to the decline is the fact that most effective antibiotics currently available are generics, with new medications often having difficulty gaining ground or showing adequate cost-effectiveness to justify a premium price [7]. Additionally, consumption of antibiotics is intentionally kept low by prescribers, for fear of breeding “super-bugs” – bacteria resistant to multiple classes of antibiotics. However, even with judicious prescribing of antibiotics, a century’s worth of experience has demonstrated a constant struggle to find new methods to combat bacteria, as bacteria evolve new mechanisms to resist current drugs. A high cost and significant technical effort is required to find new antibiotics, particularly against Gram negative bacteria [8]. Additionally, after the technical challenges are overcome, clinical trials for antibiotics can be costly and demanding as they require different trials for each new indication in varying organ systems [8], resulting in higher clinical costs than drugs in other therapeutic categories [9]. All of these factors combine to produce an inherent tension between health policy and industry research objectives. The market also fails specifically for antibiotics because the necessity for continual development of new antibiotics stems from the impending future threat of resistance, not just the current lack of efficacy. A critical market demand large enough to spur development may not exist until a crisis has emerged. The result, as shown in Figure 1, [10], is that the total number of new molecular entities of antibacterials approved annually has slowly declined over the past two decades.

Prudent health policy seeks to encourage development of additional antibiotic formulations and mechanisms, even as financial pressures make this goal less attractive to industry. To combat this problem, a US government Interagency Task Force on Antimicrobial Resistance was formed and subsequently developed an action plan in 2001 with four broad areas of focus: increased surveillance; prevention and control, research and antimicrobial development. Lack of funding from the government has led to underfunding of these mandates and subsequently little progress in any of the above areas [11]. In response to this, the Infectious Disease Society of America launched a public awareness campaign aimed at stimulating public concern and promoting effective policy concerning the growing trend of antibiotic resistance and declining novel antibiotic development. However, persistent inaction in passing appropriate federal legislation has stymied significant progress in the war on anti-microbials [11]. The STAAR Act (“Strategies to Address Antimicrobial Resistance) was introduced into Congress with the aim to build on previous efforts by the Interagency Task Force, but this did not pass Congressional approval. More recent efforts have come with the formation of the Transatlantic Task Force on Antimicrobial Resistance (TATFAR) in 2009, a collaboration between the European Union and the United States focused on addressing the problem of antibiotic resistance. TATFAR identified three key areas of focus [12]:

1. Monitoring and encouraging appropriate use of antibiotics in the medical and veterinary communities

2. Prevention of drug resistant infections

3. Developing strategies to enhance the antibiotic pipeline

In their 2011 report [12] detailing recommendations for increased collaboration between the EU and US, the difficulties of stimulating research across national borders is recognized. However, the TATFAR report strongly urges consideration of new mechanisms to incentivize research to stoke the antibiotic pipeline. One such piece of legislation designed to address these shortcomings is the *Generating Antibiotic Incentives Now* (GAIN) Act, passed in 2011. Under the GAIN Act, qualifying antibiotics will receive priority review from the Food and Drug Administration (FDA), as well as an additional five years of market exclusivity. Also, the legislation mandates a review of regulatory requirements for clinical trial data, to take into account the limited number of patients available for enrollment in the case of more rare pathogens [5,13].

The passage of the GAIN Act reflects a growing public awareness of the deepening crisis in antibiotic resistance and lack of innovation, as demonstrated in a flurry of recent news reports [14,15]. The Chief Medical Officer of England has famously compared the risk of antibiotic resistance to that of terrorism, as a significant threat to domestic safety [16]. The concept of antibiotics as a non-renewable resource has even been proposed to stimulate a shift in public perception regarding the overuse of antibiotics in self-limiting infections [17]. If this paradigm shift were to be successful, it may help reduce the prevalence of antibiotic resistance, but with the added effect of decreasing market demand for antibiotics, creating a further disincentive for investment in research. More recently, the WHO’s Consultative Expert Working Group examined various policies and incentives to promote antibiotic development. They concluded that free market competition is the best mechanism to achieve affordable new products, but this should be accompanied by a delinking of R&D costs and drug price [18].

This conclusion highlights the most vexing aspect of the antibiotic development shortage: how to simultaneously stimulate research and pay for R&D costs while ensuring affordability. Traditionally, incentives to encourage research for neglected drugs have fallen into two main types – push and pull methods- each with its own shortcomings [19]. Push mechanisms focus on moving the supply curve by decreasing the cost and burden of risk to the developer. This is accomplished with direct funding from grant making bodies, tax breaks and patent pools [20]. Unfortunately, while these mechanisms facilitate research on the front end, they have no inherent ability to further reward successful completion of a drug development project, potentially misaligning the incentives of the developers and the funders.

Pull mechanisms focus instead on moving the demand curve by increasing the incentives or revenues for successful development of a final drug or vaccine. Examples of this include the establishment of prizes or market guarantees for neglected vaccines or drugs. These focus on rewarding successful output, instead of subsidizing the initial development; however, the challenge with these mechanisms is in establishing an appropriate level of incentive (or market price) such that it spurs increased interest in development without being excessively wasteful [21]. Another challenge unique to pull mechanisms is to sufficiently specify the characteristics of a drug or vaccine that will qualify for the final award – too specific and it might deter developers, too broad and the goals of the funder may not be realized.

The size of the incentives needed are not small. Estimates of drug development capitalized costs range from $800 million to over $2 billion, depending on the class of pharmaceutical. Therefore, any mechanism to stimulate R&D must be backed by adequate funding [22-26]. Any push mechanism will require upfront investment, thus all investments will have the same opportunity cost of capital per dollar, depending on the timing of the investment. This is expected to be more efficient since smaller sums are needed earlier on, thus very early investments are smaller, and therefore comparatively less money is lost in foregone interest.

Development may also be incentivized by front loading contracts, with larger payouts to companies to develop the first drug in a particular class. This spurs initial, rapid development, but does not guarantee long term production. Instead, an appropriate commitment should balance initial incentives with long term supply needs, and encourage subsequent improvement in drug design or supply [25]. Similarly, an effective network must be built to ensure appropriate distribution of drugs to the intended recipients, thereby guaranteeing the demand vital to an appropriate return on investment by the company. This is particularly true if only a price is guaranteed, and not a specified flat payment. A specified prize for successful development of a particular entity gives a strong incentive for development, but runs the risk of purchasers paying for drugs they may not be able to practically deliver. An ideal solution would both guarantee a price, and ensure a sufficient demand.

## Methods

The combination of guaranteeing price, while ensuring demand is not easily achievable with traditional research incentive schemes. Therefore it was hypothesized that a combination of various incentives might prove more effective in accomplishing this. Recent research from the field of neglected vaccine development provides two promising and novel mechanisms to stimulate R&D, both of which hold lessons for the field of antibiotic development. The first of these mechanisms is the idea of an Advanced Market Commitment (AMC) for vaccines [27], while the second is the Call Options for Vaccines (COV) method [28].

The Center for Global Development (CGD) advocated the idea of an advanced market commitment (AMC), funded by international philanthropic agencies or NGO’s [27]. The approach by the CGD guarantees a certain price, subsidized by sponsors of the advanced market commitment, for a specified number of units of a qualifying vaccine. The decision to purchase a newly developed vaccine is undertaken by individual countries or purchasers, who contribute a small copayment, with the difference between the guaranteed price and the copayment made up by the AMC sponsors. This commitment holds for a set number of vaccines, the number of which is determined by the initial guaranteed price, such that the entire market commitment of the sponsor is US $3 billion. For instance, an international sponsor may commit to a market price of US $15 per dose for a malaria vaccine. A developing nation might be able to pay US $1 per dose, with the balance made up by the sponsor of the AMC. The first 200 million units will be guaranteed to fetch such an artificially high price, with any additional units sold at a reduced price directly to the purchasers. While the price is guaranteed for any vaccine, the quantity is not, as countries are not obligated to purchase developed vaccines. This price (but not quantity) guarantee removes the need to explicitly specify the conditions of an acceptable vaccine, but still maintains a credible commitment to purchase. This mechanism underscores the implicit assumption that the key to successful development is to translate social benefits into corporate profits.

With this in mind, it is important to understand some of the tools utilized by corporations to evaluate profitability of projects. One of these is the concept of net present value (NPV). The net present value is the current total of all expected cash flows for the life of a project minus the costs [29]. In an extremely simplified example, the NPV is the difference between an investment made at time 0 and the subsequent cash received discounted by a set rate (usually the interest rate). Variants of this method can be utilized by companies to determine whether or not a project is worth investing in [30]. If the NPV is greater than 0, after adjusting for all of the projected costs and returns, a project would be considered favorable for investment. It is thus intuitive that the ultimate decision to invest in a project can be altered in one of two ways – decrease the costs or increase the cash flow. To apply this to the previous discussion, pull mechanisms help to increase cash flow, while push mechanisms lower costs.

**Figure 2:**
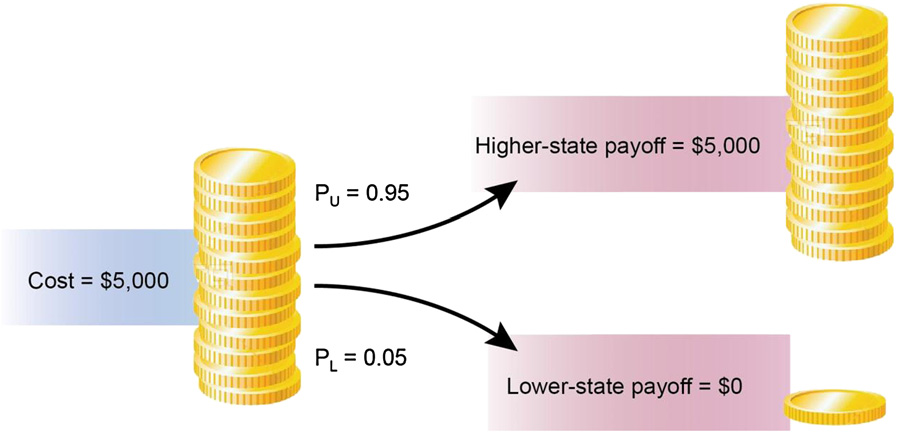
**Example of a project with a cost of $5,000 and two possible outcomes – success or failure, each with differing payouts. **Source: [28].

A caveat to the task of increasing cash flow is the uncertainty of future returns that is intrinsic to the research and development process. Thus, expected cash flows are often modeled in part based on the likelihood of payout. A simple binomial model can be used to understand the associated costs of success or failure in a single stage of development on ultimate cash payout, and subsequently NPV. Figure 2 gives an example of such a project with a cost of US$5,000 and two possible outcomes – success or failure, each with a pre-determined payout. If the project is successful, the payout is US$5,000, and if it fails the payout is US$0. Each of these states has a pre-determined probability of success, and the total valuation for the project is the sum of the products of each payout and their probabilities. In the example in Figure 2, a payout of US$5,000 would not be sufficient to induce any rational actor to undertake the project. Instead, the project can be made more attractive by increasing the higher payout or lowering the initial cost. This is a fundamental and important principle - NPV can be adjusted by altering future payouts or lowering costs (in this case by initial payments). Potential for savings exists if a payer is willing to shoulder some of the risks. The Call Options for Vaccines (COV) model, explained below, is based on this simple premise.

The COV model has been described previously [28]. However, it is worth briefly reviewing the principles of this model to inform future discussion. The COV model proposes an incentive mechanism combining both push and pull methods, based loosely on the principles of call options in equity markets. In a typical call option, an investor can purchase the right to buy a share of stock at a later date for a fixed price. The idea behind an option is that an investor can pay a premium now for the potential to profit later. However, the ability to profit later is not guaranteed, thus risk is involved in the payment of the premium. The seller of the call option also undertakes some risk, since any potential profit will come at the seller’s expense. For example, an option might be bought on a share of Stock X (which currently trades at US$10 per share) for US$1, with a strike price of US$15. If the stock price moves above US$15 per share prior to the option expiration date, the holder of the option may then purchase one share of Stock X for US$15. Therefore, three scenarios are possible:

1) The share price stays below US$15, the option is not exercised and the purchaser loses a total of US$1;

2) The share price moves to $16 at which point the option may be exercised, and a share is bought for US$15, and sold immediately for US$16. In this case, the holder of the option yields a net of US$0 (given the initial outlay of US$1). If the stock price is between US$15 and US$16 the option may be exercised, but the holder will lose money, up to a net loss of US$1;

3) The share price goes above US$16, at which point the option is exercised, the holder purchases a share of stock at US$15 and sells it immediately at the current higher market price – this results in a profit equal to the difference between the current stock price and US$16, (strike price plus initial option cost).

The COV model functions along similar lines. Instead of buying the right to purchase a stock at a future date, the purchaser buys the option to purchase a set number of pharmaceuticals at a date in the future. This can be done at virtually any stage of development, with higher option prices at later stages (given the lack of uncertainty). In no way is the purchaser guaranteed that the drug will ever make it to market, but if it does, the purchaser will be able to obtain a fixed quantity of it for a discounted price. The purchaser is not exposed to the risks and potential costs of any further development, and companies are potentially given money at crucial, earlier stages in the development process.

## Results and discussion

The principles underpinning the COV model can be adapted to the problem of stimulating antibiotic development, with a few important changes, resulting in what we have named the Options Market for Antibiotics (OMA) model. To illustrate the purchase of an antibiotic using the OMA model we will utilize an example that closely follows the one above.

In this hypothetical scenario a large pharmaceutical company may begin development of an antibiotic that holds great promise in treating a disease whose major burden is felt in developing nations. Examples of diseases that may disproportionately affect developing nations are typhoid and cholera [31]. Traditional market forces would not make marketing or testing of this antibiotic profitable, since the purchasing power of the consumers or patients in the developing nation may be relatively low. A third party interested in combating this bacteria in that nation or others may seek to stimulate development of effective antibiotics. In our options model, the third party payer, an NGO or government agency, could purchase options for the antibiotic, which they would redeem if and when the drug ever made it to market. If they purchased the options early in development, the price of the options would be low, but the risk of the drug failing may be comparatively high. If the option was purchased near the end of the product development cycle, its cost would be much higher. The purchaser of the option would benefit in two ways: first, by incentivizing development of a drug that they desire, and second, by receiving the drug at a discounted price compared to the market cost at the time of product launch. The price of the initial option may be a few pennies per dose desired (depending on the stage at which it is purchased), but the holder of the option may then receive a discount of several dollars off the ultimate purchase price. If the initial cost of the option was $0.20 per dose desired, and 1 million doses were anticipated, the third party payer may spend $200,000 up front. However, if the antibiotic ultimately passes all regulatory tests and becomes available on the market, the holder of the options may then be able to purchase it at a discount of $2 per dose (this is an arbitrary amount as the actual discount would vary based on the drug’s market cost and options cost). For the 1 million doses desired, the savings would be $1.8 million ($2 million discounted total price minus the $200,000 upfront cost of the option). Of course, a strict accounting of the savings would include an analysis of the interest lost during the development process, as well as the relative risk assumed by the purchaser. The third party could then utilize the purchased antibiotics for their own purposes, or sell the options or antibiotics to patients or health ministries for a profit. If the antibiotic never makes it to market, then the third party purchaser would lose the initial $200,000 investment.

Similarly, a “super fund” with the goal of stimulating antibiotic development in a community of developed nations, such as the EU, could purchase a similar set of options based on the perceived burden of disease. The overriding purpose of any such “super fund” would be to set priorities for funding and provide the infrastructure for appropriate evaluation of potential investments. Upon final approval of the antibiotic, this super fund could then distribute the options to buy the drug to constituent states, based on either need or contribution to the fund. If desired, this distribution of call options could be based on Ramsey pricing [32,33], such that poorer EU countries would pay comparatively less than richer EU countries. The strike price of the option would remain consistent, but the presence of the options allows for a secondary market to achieve equity based on societal or national values. This could be employed to assist distribution in the least developed countries. If an NGO were to purchase call options for these countries, they could effectively distribute the options to the countries at the time of market approval. As will be discussed below, the strike price will be set at marginal cost of production, therefore allowing those countries to purchase the drug at a minimum cost, with the cost of the effective subsidy borne by the NGO.

As with any investment decision, the efficacy of the OMA model hinges on balancing the risks and rewards of investment. This risk varies with the stage of the investment, and the rewards vary with the cost of the option, the market price of the final drug and the strike price (the cost of the drug to option holders). The difference between the final drug price and the strike price represents the value of the option to the holder at the time of redemption, and the net profit is this value minus the initial cost of the option. Mathematical models exist for pricing options in the world of finance; however, these rely on historical data about the volatility of the stock price. This is likely not to be applicable to our options model, which relies more on the decision by the company to continue development or abandon the project, at each new stage of development. Therefore, a binomial options pricing model may more accurately characterize this investment decision.

The Binomial Option Pricing Model [34] may be utilized by firms to assess whether or not to engage in research projects, based on a value maximization approach. Full details of this model can be found in Additional file 1. Briefly though, implementation of the model relies on four main variables, the value of the Call option (C), the strike price of the option (X), the initial investment required (I) and the payoff owed by the purchaser to the company for successful development, M. M would be a function of the premium placed on development of a particular drug, determined by the societal need for it. The CGD concept of the Advanced Market Commitment could play an integral role in determining the socially acceptable payout for development of a new drug. The OMA model is obtained by integrating the Advanced Market Commitment into the original COV model, through the use of a new variable, *AMC*, which represents the determined value of the market needed to stimulate research. In the CGD model, $3 billion was the AMC amount needed to incentivize development of vaccines. Along those same lines, it could also be set at EURO 1.25 billion, a figure arrived at by Towse and Sharma [35]. Ultimately the AMC amount will evolve over time depending on the regulatory and R&D costs associated with average drug development. The relationship of the determined market value for a specific drug and the individual drug’s possible project payout is given in the following equation:

$$M=\frac{\mathrm{AMC}\times Q}{N\times E}$$

Here we introduce three new variables, *Q*, *N and E*. *Q* accounts for the novelty of a drug in comparison to current alternatives, and will range in value from 0 to 1. Obviously 1 is an ideal, assigned only to a completely novel drug, addressing an as yet unmet need with superb efficacy. The value of *Q* could be determined by an independent advisory panel which would assess the likelihood of the candidate drug to contribute novel therapeutic benefit. Drugs with questionable efficacy would be scored with a lower *Q*, meaning that their intrinsic project payoff would also be lower. Similarly, *N* represents the total number of all drugs currently available in the same class. This would function to encourage development of drugs in novel therapeutic classes, as opposed to finding isomers and creating Me-Too drugs to gain marginal market share. Finally *E* represents the average efficacy of all current drugs in the same class, and can range from 0 to 1. Therefore, with these parameters, a truly unique drug with high likelihood of efficacy would yield a prize of the full advanced market commitment. A drug with little proven efficacy, entering into an already crowded field would have a much smaller market value (M), and thus less likelihood of securing a favorable return for the potential costs of development. Drugs that enter into a field marked by ineffective competitors would also fare well. This mechanism actively encourages novel drug development in neglected areas and targets financial rewards to that innovation.

Finally, the strike price (or exercise price), X, must be defined. In traditional financial call options, this price determines whether or not the option will be redeemed, since the redemption condition is whether or not the stock price reaches the strike price. However, in our model, the condition of redemption is approval of the drug (i.e. successful development). Thus, we have chosen to set the strike price as the marginal cost of production. In this way, the manufacturer of the drug does not lose money for each additional unit sold to holders of the call option; however, the cost of development should not be borne at later stages by holders of the call option (they have invested previously to support development by purchasing the call option).

Accurate information regarding the probability of success at each stage in a product’s life cycle is key for fair valuation utilizing the OMA model. DiMasi has reported the probabilities of different classes of drugs successfully transitioning from one phase of clinical trials to the next over an 11 year period (Table 1). The overall estimated success rate for anti-infectives ranged from 15.6% to a maximum of 27% [36]. Anti-infectives have a lower rate of initial success transitioning from Phase I to Phase II, but a higher rate of transitioning from Phase III to regulatory review. This brings to bear an important point, the challenge of developing antibiotics is both technical and financial. Fewer drugs are being brought to market- in the face of such high approval in the later stages, one can only surmise that this is due to lack of initial investment, as well as scientific challenges to investigation and discovery of antibiotics and not end stage regulatory hurdles.

**Table 1:** Transitional Probabilities per Development Phase from 1993–2004

**Therapeutic class**	**Phase I-II (%)**	**Phase II-III (%)**	**Phase III- RR (%)**	**RR-approval (%)**	**Clinical approval success rate (%)**
Anitneoplastic/immunologic	71.8	49.0	55.3	100	19.4
Cardiovascular	62.9	32.4	64.3	66.7	8.7
CNS	59.6	33.0	46.4	90.0	8.2
GI/metabolism	67.5	34.9	50.0	80.0	9.4
Musculoskeletal	72.4	35.2	80.0	100	20.4
Respiratory	72.5	20.0	85.7	80.0	9.9
Systemic anti-infective	58.2	52.2	78.6	100	23.9
Miscellaneous	62.8	48.7	69.8	91.3	19.5

Notes: Through to June 2009.

CNS, Central Nervous System; GI, gastrointestinal; RR, regulatory review.

Source: [36] DiMasi, J.A., et al., *Trends in Risks Associated With New Drug Development: Success Rates for Investigational Drugs*. Clin Pharmacol Ther, 2010. 87(3):272–277.

### Determining when to invest

**Figure 3:**
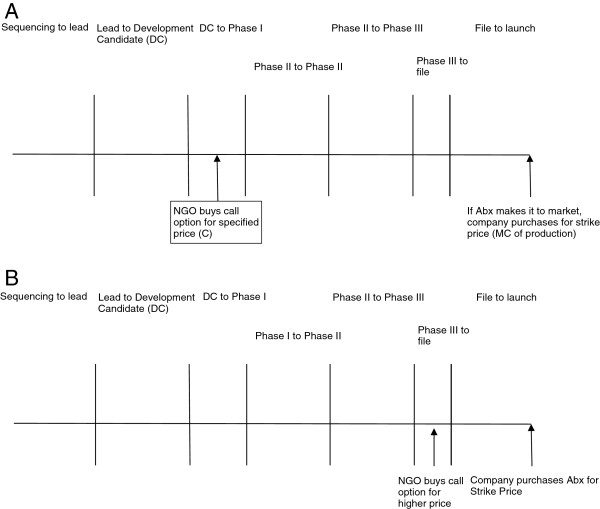
**Timeline of drug approval with corresponding examples of call options pricing. A: **Investment in early stage of development, the cost of the call option is low, but the risk is high. **B:** Investment in late stage of development, the cost of the call option is high, but the risk is low.

One of the benefits of the OMA model is that it allows potential investors to adjust their exposure to risk based on the phase of development in which they choose to invest. Greater savings may be had by investing early, but this will accompanied by substantial risk (Figure 3A). However, the low cost may allow a purchaser to effectively seed a number of projects for a relatively small amount of money, thereby mitigating some of the risks. Later phase investments will be relatively safer as the antibiotic nears completion of all regulatory hurdles, but the savings will be subsequently minimal In fact, if the call option is fairly priced, as it enters the final phase of testing it will reflect nearly the market cost of the soon to be approved drug (Figure 3B). This highlights the versatility of this model to a wide array of potential purchasers with different interests. Realistically, most parties will invest in a stage somewhere between the two highlighted above.

It should be noted that this mechanism may prove most useful for small and medium size enterprises, which may have a comparatively high cost of capital, as opposed to well capitalized large pharmaceutical firms. It could also act as a salvage mechanism to rescue promising compounds which were abandoned due to lack of financial incentives. The intellectual property of these salvage compounds could be purchased by firms, and made profitable by infusion of funds via the OMA model.

The impact of push mechanisms or investment at early stage cannot be overstated. Spellberg et al. [37] recently demonstrated that an early stage push incentive may be 95% smaller than an equally effective pull mechanism, simply due to the effects of time discounting. In essence, timing matters in R&D and judiciously applied incentives at appropriate time intervals may have big effects on stimulating research.

### Further considerations

The OMA model depends crucially on the free exchange of information between potential purchasers and developers. Access to all results from animal testing and preliminary clinical trials would be essential to allow proper evaluation of the viability and efficacy of the drug. This sort of evaluation would require a multi-disciplinary team with representatives suited to evaluate the medical efficacy, pharmacologic profile and cost effectiveness of purchasing options. Corporations may prove hesitant to disclose such sensitive data, but appropriate steps could be taken to ensure confidentiality.

Of course attention to intellectual property (IP) concerns should always be considered when developing a new incentive strategy. The European Institute for Innovative Medicines, a public-private partnership to promote collaboration between industry and academics to aid the drug development process [38], provides a cautionary tale on the impact of IP concerns in stimulating or prohibiting collaboration. Since its inception in 2007, the initiative has been plagued by concerns over ownership of IP, particularly from academic groups. The most recent projects focus on enabling the development of new antibiotics by creating a pan-European clinical trial network and exploring methods to fight multi-drug resistant pathogens through a combined EUR €224 million grant. Despite this ambitious effort, concerns over IP sharing still remain [39], however, necessary efforts are being made to promote greater transparency in clinical trial data stemming from this partnership [40].

In our proposal, if new IP is developed during a project in which options are purchased, it would be paramount that a portion of any subsequent dividends stemming from that IP be shared with the holders of the initial options. This would be crucial to prevent companies from ending projects in late stages of development to avoid redemption of call options, and then utilizing that same intellectual property to develop similar drugs where no options have been purchased.

It is possible that promising drugs might be conceived by companies ill-equipped to carry their development through. This must be taken into account when making the decision to invest - thus the need for a multi-disciplinary group to evaluate potential projects. It would rely heavily on full disclosure of all relevant documents. International purchasers would need to hire people with specific skills in financing, project valuation and/or real options valuation (ROV) assessment. However, it is reasonable to believe that if information is shared appropriately, the skills of the purchasers could closely match those of the drug developers in this area. While risk remains to purchasers, this risk is balanced by the high priority of stimulating research in this area.

This model is not without risk to the developer as well, as additional hidden costs may manifest at the time of final regulatory approval, possibly in the form of additional clinical trials or testing. This risk could be modeled and priced into the call option, or additional contingency funds could be set aside, potentially paid for by the purchaser. If difficult barriers to marketing approval remain, alliances could be formed between firms with complementary capabilities to enhance their overall competitive advantage. This would increase the chance of the drug making it through the final stages of regulatory approval as alliances have been previously shown to be more effective in drug development than single institutions [41].

A valid critique of the model is that the premium price of any antibiotic outside of the discounted price may be substantial, but we believe this would only encourage purchasers to participate in this scheme for future developments. Summation of the prices for the discounted and non-discounted drugs could still result in an overall global savings, but even if the overall costs are even, the plan still has merit in its ability to encourage innovation by providing early seed money. On the flip side, one can imagine professional antibiotic investors who purchase the options rights then sell to NGO’s for a profit. If in the end the cost savings are minimal, but innovation is accomplished we would consider this successful. Real options modeling is a well established field that helps pharmaceutical companies make development decisions, we are trying to harness this modeling to aid in development by combining both initial incentives for development while still stimulating delivery of a final product. The idea is to create a sophisticated risk sharing model between all stakeholders.

## Conclusions

It is clear that innovation in technical development as well as regulatory and policy schemes are crucial to ensure a full pipeline of effective antibiotics. The current market pressures, combined with unique properties intrinsic to the method of use of antibiotics, conspire to create many imperfections that prevent aggressive investment. Nevertheless, the attention given to the lack of new antibiotic development in the lay and scientific press has stimulated some momentum in addressing this problem. The launch of the IMI projects in the European Union coincides with the passage of the GAIN Act in the US. These efforts are designed to lower the cost and regulatory requirements for antibiotic development. Another innovative financing project is the Health Impact Fund (HIF), designed to incentivize neglected drug development. The HIF seeks to establish a global fund which will register new medicines designed for neglected diseases and reward companies according to the global burden of disease that each drug alleviates, as measured in QALY’s. Companies, in exchange for the reward payments during the first 10 years of marketing, will then manufacture and distribute the drug at cost during this time period. At the end of the first decade, the companies must agree to allow generic manufacturing of the drug [42]. This certainly poses a number of practical challenges, not the least of which is assessing the health impact of each drug.

Despite these technical challenges, it is clear that a hybrid of push and pull mechanisms will be necessary to effectively stimulate research in this field [43]. Many such proposals have been put forth, and we add to this the Options Market for Antibiotics. The distinguishing feature of this scheme is that it seeks to incentivize development early on, while sharing risks between developers and payers. The goal of the OMA is to allow the market to function effectively at different points in a drug’s life cycle, instead of simply at the time of marketing. The OMA model effectively takes the subsidy proposed by the AMC, and transfers that to companies at earlier stages, appropriately discounting it for the time value of money, as well as the risk assumed. The underlying tenets of the need for a subsidy are the same; however, this hybrid mechanism simply advocates this subsidy at different stages in the product life cycle.

As pharmaceutical companies continue to search for blockbuster drugs to be sold at a premium price, attention must be paid to the continued research and development of vital but less profitable antibiotics, vaccines and other neglected pharmaceuticals. Much work has been focused on this in the past with mixed success. Now, as newer models to stimulate R&D are developed, a more critical examination of the risks and benefits must ensue.

## Abbreviations

AMC: Advanced Market Commitment; COV: Call Options for Vaccines; CGD: Center for Global Development; FDA: Federal Drugs Administration; HIF: Health Impact Fund; IP: Intellectual Property; NPV: Net present value; NME: New molecular entities; OMA: Options Market for Antibiotics; TATFAR: Transatlantic Task Force on Antimicrobial Resistance.

## Competing interests

Neither Dr. Brogan nor Professor Mossialos or their immediate families have any competing or conflicting financial interests related to the scope of this work.

## Authors’ contributions

DB and EM drafted the paper jointly. Both authors read and approved the final manuscript.

## Supplementary Material

Additional file 1 **Source: **[34]** Adapted from Cheng, J. 2006.** Click here for file
