# Supplementary material for: Incentives for new antibiotics: the Options Market for Antibiotics (OMA) model
Source: Global Health. 2013 Nov 7;9:58. doi: 10.1186/1744-8603-9-58 (PMC4226193; doi:10.1186/1744-8603-9-58)
Supplement: Additional file 1 — Source: [34] Adapted from Cheng, J. 2006. [file 1744-8603-9-58-S1.docx]

In the binomial model, a call option can be priced using the following equation:

$$C=\frac{S_{u}\times R-S_{u}\times d-X\times R+X\times d}{(u-d)\times R}$$

In this equation, the above variables represent the following:

C = the value of the call option

$S_{u}$ = the value of the stock of the company if the project succeeds

$S_{d}$ = the value of the stock of the company if the project fails

X = strike price of the option (the price of the stock at which the option would be redeemed)

R = (1 + risk free interest rate)^T (years)^ (essentially accounting for the time value of money)

u = $\frac{S_{u}}{S}$

d = $\frac{S_{d}}{S}$

S is the current value of the company after the project is undertaken but before the final outcome is known. It is essentially an average of the value of each state of success or failure based on their respective probabilities. It can be mathematically represented by:

$$S= P_{u}\times S_{u}+P_{d}{\times S}_{d}$$

In this equation, $P_{u}$represents the probability of the project succeeding (achieving the up state), while $P_{d}$represents the probability of achieving the down state (failure). These will necessarily be a function of the project’s current state of development. The nature of pharmaceutical development is such that each independent stage carries dramatically different risks of success or failure. The success of the overall project can be viewed as the probability of success of a series of independent events. We must assume that the chance of success in any given stage is independent of the probability of success of any future stage. Also, success in past stages has no effect on future probabilities, other than success being a precondition to continue moving forward. Therefore, the probability of success from any given stage is equal to the product of the probabilities of success for all future stages:

$$P_{u}=\prod_{i=m}^{n} P_{i}$$

where m is the current stage of development, n is the final stage of development and $P_{i}$ is the probability of success for any given stage. Conversely, the probability of the project reaching the down state, $P_{d}$, is simply:

$$P_{d}=1- P_{u}$$

The value of the company if the project succeeds can be obtained from the following:

$$S_{u}= {(S}_{0}- I) \times R+M$$

This effectively states that the net increase in the company’s value after the initial investment required for the project, *I*, will be equal to the payoff *M* from the project’s revenues at some future date. In our applications, *M* would be a function of the payoff deemed necessary by society to stimulate development for a particular class of drugs.

$$M=\frac{AMC\times Q}{N\times E}$$

*Q* is a parameter measuring the efficacy and novelty of the drug when compared to its peer group, and will range in value from 0 to 1. N is the number of drugs in the same therapeutic class that are currently available. *E* represents the average efficacy of all current drugs in the same class, and can range from 0 to 1. AMC is the societal value for a particular class of drugs.

The company value in the down state (project failure) must also be defined, and follows from the above equations to be:

$$S_{d}= {(S}_{0}- I) \times R$$
